# Metagenomic analysis to identify novel infectious agents in systemic anaplastic large cell lymphoma

**DOI:** 10.1186/s13027-021-00404-0

**Published:** 2021-11-14

**Authors:** Parag Mahale, Jason Nomburg, Joo Y. Song, Mia Steinberg, Gabriel Starrett, Joseph Boland, Charles F. Lynch, Amy Chadburn, Paul G. Rubinstein, Brenda Y. Hernandez, Dennis D. Weisenburger, Susan Bullman, Eric A. Engels

**Affiliations:** 1 Infections and Immunoepidemiology Branch, Division of Cancer Epidemiology and Genetics, National Cancer Institute, Rockville, MD USA; 2 Department of Medical Oncology, Dana-Farber Cancer Institute, Harvard Medical School, Boston, MA USA; 3 Department of Pathology, City of Hope National Medical Center, Duarte, CA USA; 4 Cancer Genomics Research Laboratory, National Cancer Institute, Rockville, MD USA; 5 Laboratory of Cellular Oncology, Center for Cancer Research, National Cancer Institute, Bethesda, MD USA; 6 Department of Epidemiology, The University of Iowa College of Public Health, Iowa City, Iowa, USA; 7 Pathology and Laboratory Medicine, Weill Cornell Medical College, New York, NY USA; 8 Stroger Hospital of Cook County, Ruth M. Rothstein Core Center, Rush University Medical Center, Chicago, IL USA; 9 University of Hawaii Cancer Center, Honolulu, HI USA; 10 Human Biology Division, Fred Hutchinson Cancer Research Center, Seattle, WA USA

**Keywords:** Lymphoma, Viruses, Metagenomics, Immunosuppression

## Abstract

Systemic anaplastic large cell lymphoma (ALCL) is a rare CD30-expressing T-cell non-Hodgkin lymphoma. Risk of systemic ALCL is highly increased among immunosuppressed individuals. Because risk of cancers associated with viruses is increased with immunosuppression, we conducted a metagenomic analysis of systemic ALCL to determine whether a known or novel pathogen is associated with this malignancy. Total RNA was extracted and sequenced from formalin-fixed paraffin-embedded tumor specimens from 19 systemic ALCL cases (including one case from an immunosuppressed individual with human immunodeficiency virus infection), 3 Epstein-Barr virus positive diffuse large B-cell lymphomas (DLBCLs) occurring in solid organ transplant recipients (positive controls), and 3 breast cancers (negative controls). We used a pipeline based on the Genome Analysis Toolkit (GATK)-PathSeq algorithm to subtract out human RNA reads and map the remaining RNA reads to microbes. No microbial association with ALCL was identified, but we found Epstein-Barr virus in the DLBCL positive controls and determined the breast cancers to be negative. In conclusion, we did not find a pathogen associated with systemic ALCL, but because we analyzed only one ALCL tumor from an immunosuppressed person, we cannot exclude the possibility that a pathogen is associated with some cases that arise in the setting of immunosuppression.

**Supplementary Information:**

The online version contains supplementary material available at 10.1186/s13027-021-00404-0.

## Introduction

Systemic anaplastic large cell lymphoma (ALCL) is a rare CD30-expressing T-cell non-Hodgkin lymphoma (NHL) that comprises approximately 2% of all NHLs in adults [1]. Risk of systemic ALCL is markedly increased among immunosuppressed people, such as those with human immunodeficiency virus (HIV) infection and solid organ transplant recipients [2]. This increased risk in immunosuppressed populations suggests a viral etiology, because risk is similarly increased in immunosuppressed individuals for other lymphomas that are caused by Epstein-Barr virus (EBV), such as diffuse large B-cell lymphoma (DLBCL), Burkitt lymphoma, and Hodgkin lymphoma, as well as for other virus-related cancers [3]. EBV might be a plausible candidate as a cause of ALCL, but most reported ALCL tumors are EBV-negative [4].

There is a general lack of evidence regarding a possible role for infection in the etiology of systemic ALCL. Sequencing tumor tissue samples can provide a wealth of information about the biology of cancer, and while most sequences are human in origin, this approach can also detect the presence of novel viral agents. This approach was used successfully, for instance, in the identification of Merkel cell polyomavirus as the etiologic agent of Merkel cell carcinoma [5]. Metagenomics is the study of the pooled genetic material (genomes) from a mixed community of organisms. When applied to a human tissue specimen, the term can refer to the characterization of any microbial sequences among the much larger number of human sequences.

In the present study, we conducted a metagenomic analysis of systemic ALCL tumor RNA to assess for the presence of a known or novel pathogen in such cases, to help determine whether an infection may be implicated in the etiology of systemic ALCL.

## Methods

Detailed methods are described in the Additional file 1.

Given the rarity of systemic ALCL (frequently referred to below simply as ALCL, for brevity), we obtained formalin-fixed paraffin-embedded (FFPE) tumor tissue from cases archived in the tumor tissue repositories of the Hawaii and Iowa cancer registries (N = 29). In addition, we leveraged a prior linkage of the Iowa cancer registry to the US solid organ transplant registry to identify DLBCL tumors in their repository occurring in transplant recipients (N = 3) as controls likely to be EBV-positive [6]. We obtained breast cancer cases from the Iowa cancer registry repository (N = 5) as negative controls because breast cancer does not have an established viral etiology. FFPE tissue from two additional ALCL cases in HIV-infected persons from Cook County Hospital (Illinois) and Weill Cornell Medicine (New York) were subsequently identified and included.

Four-micron tumor sections on charged slides were obtained for all ALCL and DLBCL tumors for histopathology and testing for EBV-encoded small RNAs (EBERs). One case (ID #IA25) that was originally reported as ALCL in the Iowa Cancer Registry was found to be misclassified DLBCL based on our pathology review. We reclassified this case as DLBCL and retained it in the study as an EBV-negative case (it was determined to be EBER-negative).

RNA was extracted from FFPE specimens, and total RNA libraries were prepared using the KAPA RNA HyperPrep Kit (Roche) and sequenced on a HiSeq 2500 platform (Illumina) (Additional file 1: Table S1, Supplementary Methods). Due to fragmentation of the RNA, libraries could be successfully prepared for only 19 of the 31 ALCL cases (61%, including only one HIV-infected case), 4 DLBCL controls (3 that were EBER-positive, plus IA25 which was EBER-negative), and 3 breast cancer controls, and these specimens were included in the metagenomic analysis.

A stepwise approach (Additional file 1: Fig. S1) was used for metagenomic classification of RNA-seq reads [7]. First, the Genome Analysis Toolkit (GATK)-PathSeq algorithm was used to perform computational subtraction of human reads, followed by alignments of residual reads to microbial reference genomes using BWA-MEM [8, 9].

To address the possibility that a known or novel pathogen was present in the reads that remained unclassified following GATK-PathSeq, we used the Viral Identification and Discovery (virID) pipeline in two approaches [7]. First, in assembly mode, the reads were assembled de novo into longer sequences (contigs) using rnaSPADES [10]. Contigs were taxonomically assigned using MegaBLAST [11], which aligns contigs with the NCI nucleotide “nt” reference database, and DIAMOND [12], which translates each contig into amino acid sequences and searches them against the RefSeq protein database [7]. Reads that were not assembled into assigned contigs in assembly mode were then analyzed with virID in read-mode (Supplementary Methods).

As a second alternative approach, all GATK-PathSeq unassigned reads were further assessed using a “kmer enrichment” strategy to identify non-repetitive 20 base pair sequences (20mers) present in at least two ALCL samples, none of the control tumors, and at least once in the RefSeq viral database [7]. We hypothesized that if a novel virus is associated with ALCL, then it would share 20mers with ALCL cases and other viral sequences, but not the control tumor specimens.

## Results and discussion

Demographic and immunophenotypic characteristics of cases and controls are provided in Additional file 1: Table S2. Briefly, a majority of ALCL cases were ≤ 40 years of age at diagnosis (N = 10; 53%), men (N = 10; 53%), White (N = 16; 84%), and diagnosed in 2010 or earlier (N = 13; 68%). All cases tested negative for B-cell markers (CD20 or PAX5), all expressed CD30, and most expressed ALK1 (N = 11; 58%) and T-cell markers (CD2 or CD3; N = 10; 53%). All ALCL cases tested negative for EBER, whereas the three DLBCL controls occurring in transplant recipients were EBER-positive.

**Fig. 1:**
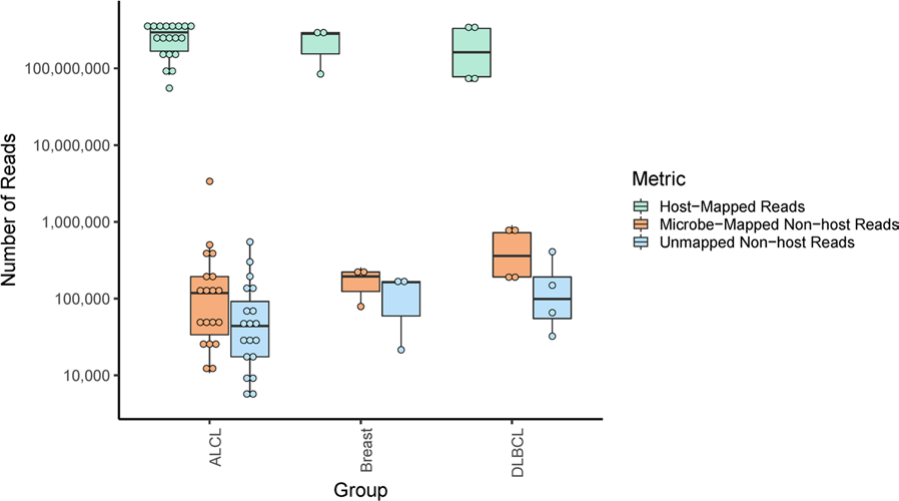
GATK-PathSeq metrics. This figure presents the number of host (human) and non-host pathogen reads that were mapped by GATK-PathSeq and the non-host reads that remained unmapped. The number of reads was plotted as box plots on the y-axis and were divided into three groups: ALCL cases, DLBCL controls, and breast cancer controls. ALCL, anaplastic large cell lymphoma; DLBCL, diffuse large B-cell lymphoma

Additional file 1 also provides information on the input RNA and the total number of RNA reads for each specimen. Across the samples, the vast majority (99.8%) of RNA reads were human in origin, as expected. Using "approach 1" in Additional file 1: Fig. S1, a median of 69.5% of the remaining reads were assigned to known microorganisms (Fig. 1). Most classified reads were bacterial, except for the three EBER-positive DLBCL controls and one ALCL case (ID# IA16) where a high proportion of viral reads were observed (Additional file 1: Fig. S2A). Hierarchical clustering of samples by their relative abundance of identified bacterial genera failed to classify the samples into specific tumor groups (Additional file 1: Fig. S3A, B).

**Fig. 2:**
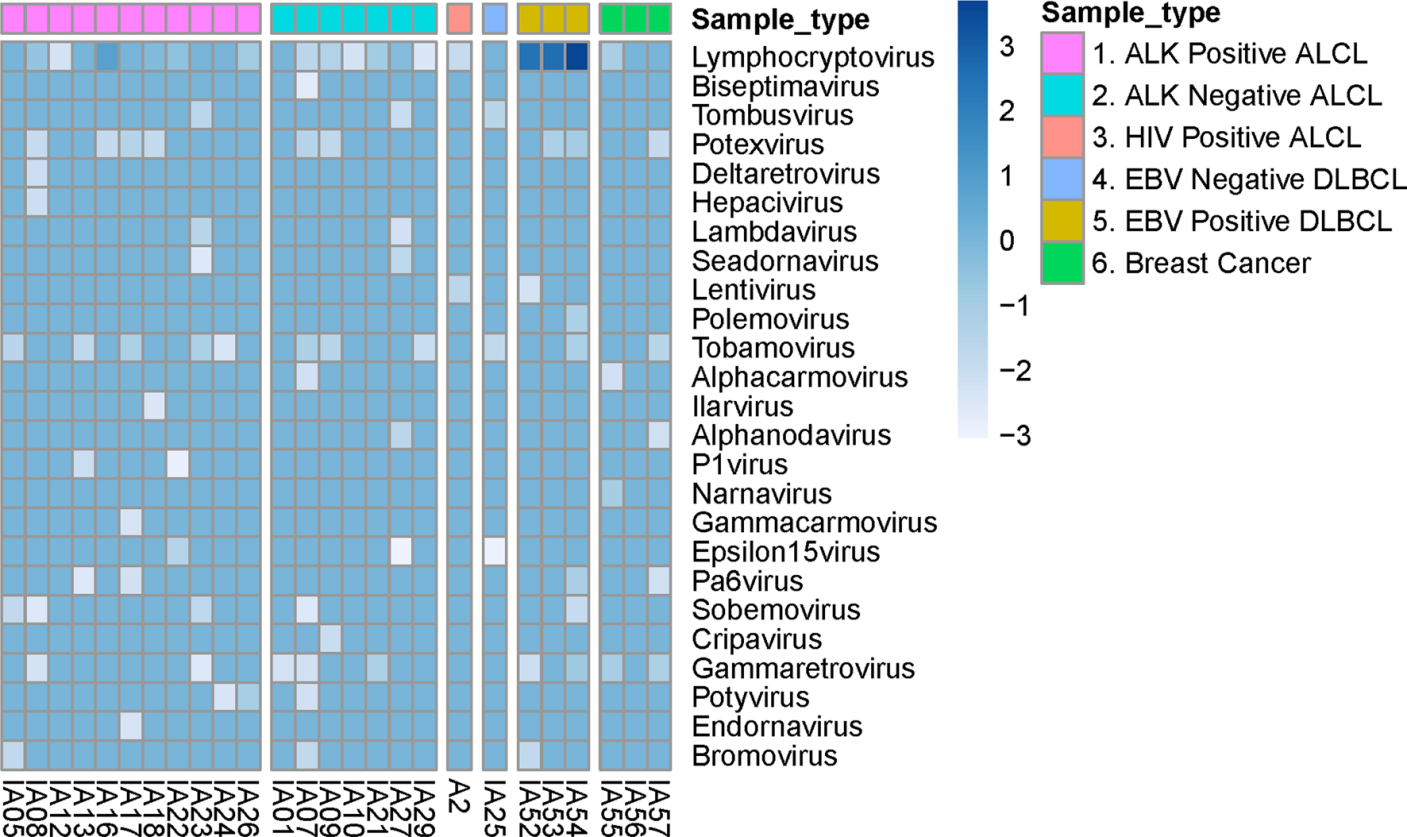
GATK-PathSeq analysis of ALCL tumors. This figure shows the heat map GATK-PathSeq viral-mapped reads at the genus level. The units used are log_10_ reads per million human reads. Samples are grouped on the x-axis as ALK-positive ALCL, ALK-negative ALCL, HIV-positive ALCL, EBER-negative DLBCL, EBER-positive DLBCL, and breast cancer. Viral genera identified are listed on the y-axis. ALCL, anaplastic large cell lymphoma; ALK, anaplastic lymphoma kinase; DLBCL, diffuse large B-cell lymphoma; EBV, Epstein-Barr virus; HIV, human immunodeficiency virus

GATK-PathSeq-based taxonomical classification did not identify any known viral genera preferentially associated with ALCL (Fig. 2). Of note, GATK-PathSeq mapped thousands of reads in the EBER-positive DLBCLs to the genus *Lymphocryptovirus*, which includes *human gammaherpesvirus 4* (EBV) and did not map reads to this genus in the EBER-negative DLBCL (ID# IA25) or breast cancer specimens. We identified a small number of EBV reads (n = 20) in ALCL case ID #IA16, which was EBER-negative, possibly due to contamination of the tumor specimen, presence of tumor-infiltrating lymphocytes containing EBV, or perhaps presence of a defective EBV genome within the tumor [13].

As shown in Fig. 2, there were many additional mapped viral genera among the RNA reads. However, these were distributed among both case and control groups, and/or the number of reads was very low (0–1 reads/million human reads). Furthermore, we observed that some virus genera with very few reads have plant hosts (e.g., *Polemovirus, Potexvirus, Ilarvirus*) or invertebrate hosts (e.g., *Seadornavirus* and *Tombusvirus*). Together, these observations suggest some degree of environmental contamination during tissue processing, storage, RNA extraction, library preparation, or sequencing [14–16], rather than a novel viral cause of ALCL.

**Fig. 3:**
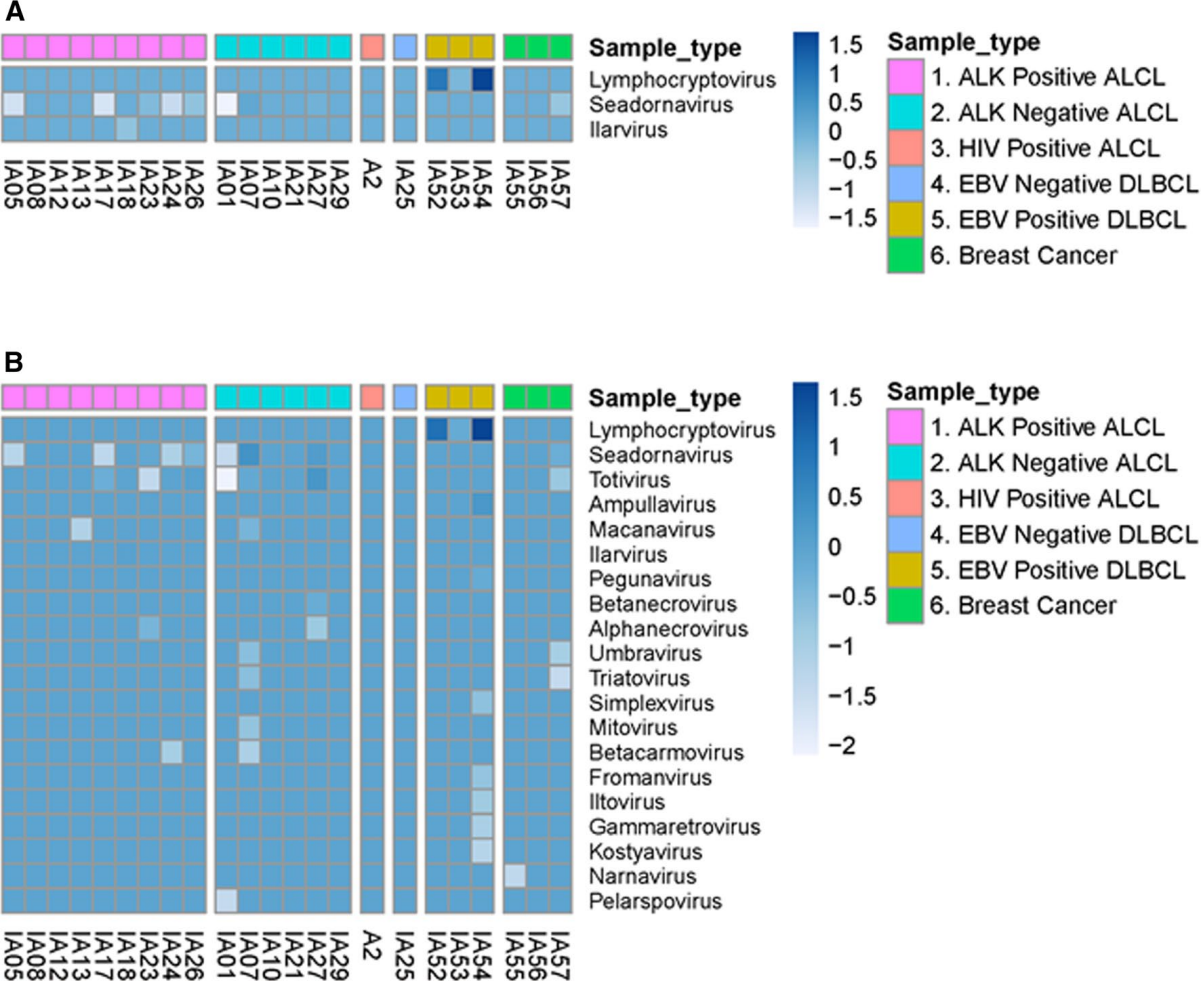
Application of virID pipeline to identify viral sequences associated with ALCL. This figure highlights the findings of applying virID algorithm to classify unmapped reads following GATK-PathSeq using the virID assembly-based approach. **A** and **B** represent the taxonomical classification of reads into viral genera after subjecting the contigs to nucleotide (MegaBLAST) and translated amino acid (DIAMOND) searches against their respective reference databases. The units used are log_10_ reads per million human reads. Samples are grouped on the x-axis as ALK-positive ALCL, ALK-negative ALCL, HIV-positive ALCL, EBER-negative DLBCL, EBER-positive DLBCL, and breast cancer. Viral genera identified are listed on the y-axis. ALCL, anaplastic large cell lymphoma; ALK, anaplastic lymphoma kinase; DLBCL, diffuse large B-cell lymphoma; HIV, human immunodeficiency virus

In additional analyses of the RNA sequence data, the assembly and read-based modes of virID did not identify previously known or novel viruses in ALCL samples (Fig. 3, Additional file 1: Fig. S4A, B). Both approaches, however, again successfully identified viral reads belonging to the *Lymphocryptovirus* genus in at least one EBER-positive DLBCL specimen. To determine if any remaining but unassigned reads originated from repetitive human sequences, such as ribosomal sequences, we profiled the remaining reads with RepeatMasker. Of the reads that were not assigned to the human or microbial reference databases by GATK-PathSeq, a median of 19.7% remained unassigned following analysis with virID and RepeatMasker (Additional file 1: Fig. S2B), supporting that our analysis pipeline was able to identify and classify the substantial majority of microbial reads.

Finally, as an alternative approach ("approach 2" in Additional file 1: Fig. S1), we conducted a BLASTN search of reads containing enriched 20mers from ALCL samples and identified Hubei tombus-like virus 8 and Norway luteo-like virus 4. However, these viruses have invertebrate hosts and are possibly environmental contaminants (Additional file 1: Fig. S5A, B) [14].

We leveraged two population-based cancer registries to identify and obtain FFPE blocks from systemic ALCL cases, which enabled us to study this rare malignancy. An additional strength of our study was our detailed phenotyping of the tumors to confirm the ALCL diagnosis and the absence of EBV. Our reliance on archived FFPE blocks that were stored for several years resulted in highly fragmented RNA that likely reduced our sensitivity, and this may also have contributed to the number of contaminating reads. Nonetheless, we demonstrated the sensitivity of our sequencing and bioinformatics approach by successfully identifying EBV reads in all three EBER-positive control DLBCL specimens, which would suggest high overall sensitivity. We did not identify EBV or other novel viruses in breast cancer specimens that we used as negative controls.

Because we analyzed only one ALCL tumor from an HIV-infected person, we cannot exclude the possibility that a pathogen is associated with some ALCL cases that arise among immunosuppressed people. A small number of EBV-positive ALCL cases have been described in people living with HIV and solid organ transplant recipients [17–19]. ALCL can resemble other T-cell lymphomas, and differential diagnosis among these subtypes is challenging. In particular, ALK1-negative ALCLs must be distinguished from extranodal NK/T-cell lymphomas, which are strongly associated with EBV infection. ALCLs exhibit characteristic “hallmark” cells, and tumor cells show diffuse and strong CD30 expression. In contrast, CD30 expression in extranodal NK/T-cell lymphoma is usually more variable. ALCLs can be positive for CD4 (which is not typically seen with extranodal NK/T-cell lymphoma), and many null-type ALCLs lack CD3 (whereas cytoplasmic CD3 expression is retained in extranodal NK/T-cell lymphoma).

In conclusion, in the first metagenomic analysis of systemic ALCLs, we did not find a pathogen that was associated with this rare cancer. Our study had a modest sample size, and further studies are required to characterize the metagenome of systemic ALCLs, especially cases arising in immunosuppressed people.

## Supplementary Information

**Additional file 1: Figure S1**. Schematic of the computational approach used in the metagenomic analysis of ALCL. **Figure S2**. Additional GATK-PathSeq analysis of tumor specimens. **Figure S3**. Detailed taxonomic classification of GATK-PathSeq assigned non-human reads. **Figure S4**. Use of read-based approach to identify viral sequences associated with ALCL. **Figure S5**. The kmer enrichment approach to identify pathogen reads from unmapped GATK-PathSeq non-human reads. **Table S1**. Quality control results for cases included in the analysis. **Table S2**. Demographic and immunophenotypic features of cases and controls. **Table S3** Immunohistochemistry and in situ hybridization panel performed on ALCL and DLBCL tumor specimens.

## Data Availability

RNA sequencing data are available in the database of Genotypes and Phenotypes (dbGaP) under accession number phs002064.v1.p1.

## Abbreviations

ALCL: Anaplastic large cell lymphoma
DLBCL: Diffuse large B-cell lymphoma
EBV: Epstein-Barr virus
EBER: EBV-encoded small RNAs
FFPE: Formalin-fixed paraffin-embedded
GATK: Genome Analysis Toolkit
HIV: Human immunodeficiency virus
NHL: Non-Hodgkin lymphoma
