# Supplementary material for: Metagenomic analysis to identify novel infectious agents in systemic anaplastic large cell lymphoma
Source: Infect Agent Cancer. 2021 Nov 14;16:65. doi: 10.1186/s13027-021-00404-0 (PMC8591940; doi:10.1186/s13027-021-00404-0)
Supplement: Supplementary file 1 — Additional file 1: Figure S1. Schematic of the computational approach used in the metagenomic analysis of ALCL. Figure S2. Additional GATK-PathSeq analysis of tumor specimens. Figure S3. Detailed taxonomic classification of GATK-PathSeq assigned non-human reads. Figure S4. Use of read-based approach to identify viral sequences associated with ALCL. Figure S5. The kmer enrichment approach to identify pathogen reads from unmapped GATK-PathSeq non-human reads. Table S1. Quality control results for cases included in the analysis. Table S2. Demographic and immunophenotypic features of cases and controls. Table S3 Immunohistochemistry and in situ hybridization panel performed on ALCL and DLBCL tumor specimens. [file 13027_2021_404_MOESM1_ESM.docx]

**Additional file 1**

| **Title** | **Page No.** |
| --- | --- |
| **Additional file 1: Figure S1.** Schematic of the computational approach used in the metagenomic analysis of ALCL ......................................................  **Additional file 1: Figure S2.** Additional GATK-PathSeq analysis of tumor specimens……………………………………………………………………  **Additional file 1: Figure S3.** Detailed taxonomic classification of GATK-PathSeq assigned non-human reads ……………...................................  **Additional file 1: Figure S4.** Use of read-based approach to identify viral sequences associated with ALCL …………….................………………  **Additional file 1: Figure S5.** The kmer enrichment approach to identify pathogen reads from unmapped GATK-PathSeq non-human reads ....….  **Additional file 1: Table S1.** Quality control results for cases included in the analysis.......................................................................................................  **Additional file 1: Table S2.** Demographic and immunophenotypic features of cases and controls…………………………….................……………….  **Additional file 1: Table S3.** Immunohistochemistry and in situ hybridization panel performed on ALCL and DLBCL tumor specimens .........................  **Supplementary Methods** ………………………………..................………...  **Additional references for the Supplementary Methods** ........................... | **2**  **3**  **4**  **6**  **8**  **9**  **10**  **12**  **13**  **18** |

**Additional file 1: Figure S1.** Schematic of the computational approach used in the metagenomic analysis of ALCL

Raw RNA sequencing reads were first processed with GATK-PathSeq, which removes all host-mapped reads and filters low quality, low complexity, duplicate reads. GATK-PathSeq taxonomically classifies non-human reads that mapped to known pathogen databases. Reads that remained unmapped following GATK-PathSeq were processed through virID in assembly mode and reads that remained unassigned were subsequently processed through virID in read-based mode. Any reads that remained unassigned were processed with RepeatMasker to determine if they mapped to repetitive human sequences. An auxiliary approach was also taken, where GATK-PathSeq unassigned reads were subjected to kmer enrichment as described in the methods and reads containing enriched 20mers were analyzed using BLASTN.

The output files from these analyses, and the code to plot the figures in the paper, are archived on Zenodo at <https://doi.org/10.5281/zenodo.4660290>.

**Abbreviations:** ALCL, anaplastic large cell lymphoma; GATK, Genome Analysis Tool Kit


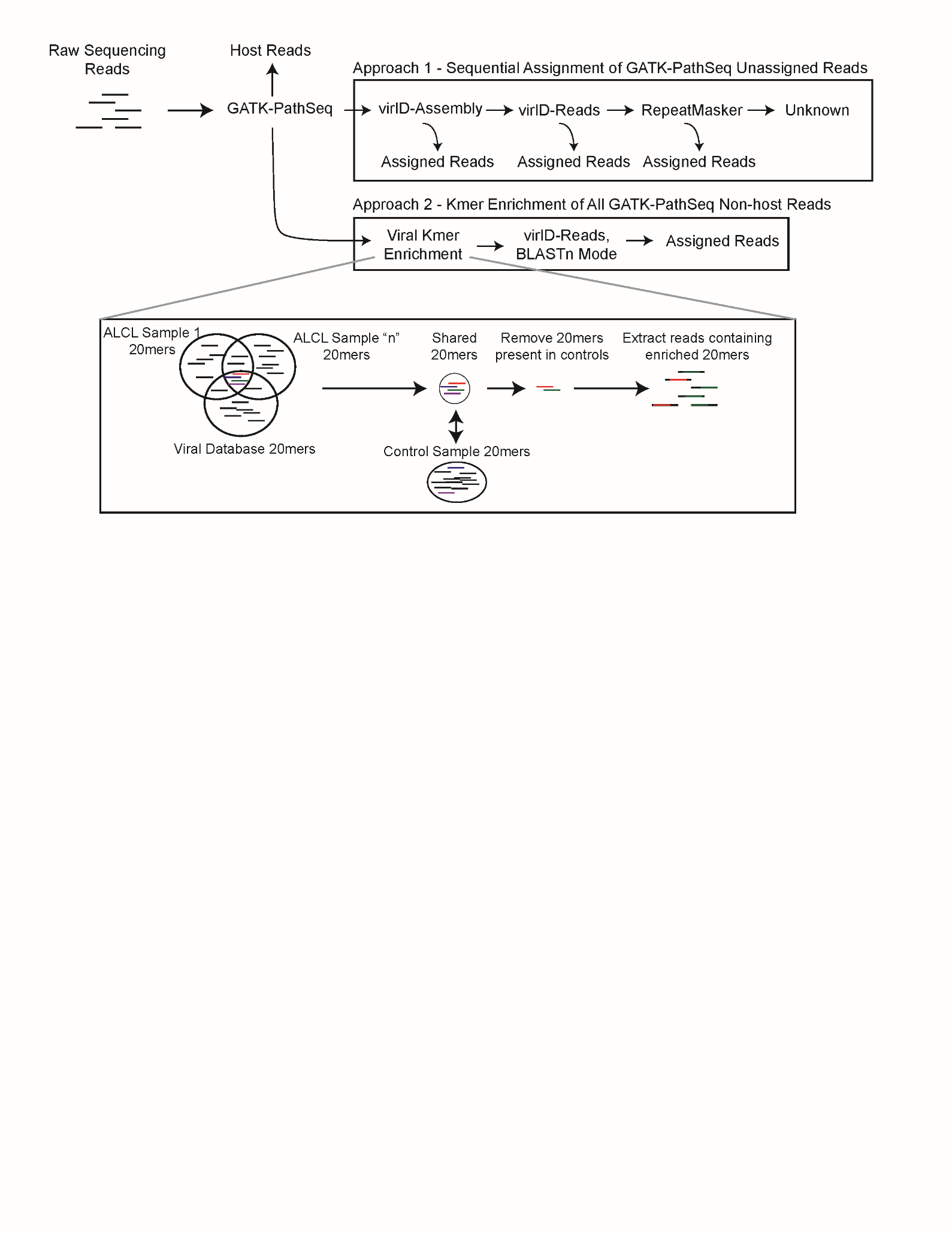


**Additional file 1: Figure S2.** Additional GATK-PathSeq analysis of tumor specimens

**Panel (A)** shows the distribution of GATK-PathSeq mapped non-human microbial reads by superkingdom (Bacteria, Eukaryota, or Viruses). The percentage of microbial mapped reads are plotted on the y-axis for each case/control specimen. **Panel (B)** shows the distribution of unassigned reads remaining after each step of the metagenomic analysis. The number of reads was plotted as box plots on the y-axis and were divided into three groups: ALCL cases, DLBCL controls, and breast cancer controls.

**Abbreviations:** ALCL, anaplastic large cell lymphoma; DLBCL, diffuse large B-cell lymphoma; GATK, Genome Analysis ToolKit.


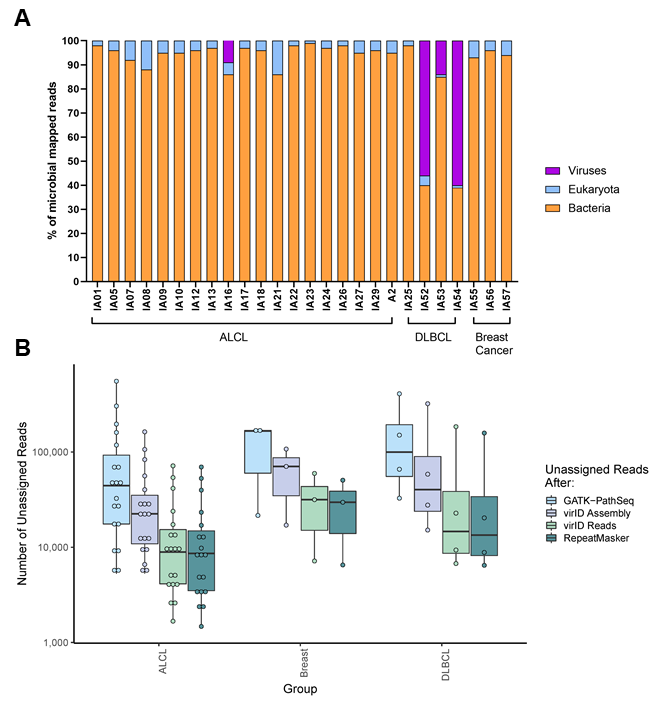


**Additional file 1: Figure S3.** Detailed taxonomic classification of GATK-PathSeq assigned non-human reads.

**Panel (A)** shows the heat map for taxonomical classification of GATK-PathSeq mapped non-host reads where the top 50 mapped genera are plotted. Most mapped genera are bacterial and are distributed among both cases and controls. The units used are log_10_ reads per million human reads. **Panel (B)** shows the genera relative abundance of GATK-PathSeq assigned bacteria-assigned reads. Top 50 genera are plotted. Samples are clustered with Euclidean distance based on the relative abundance of these genera. In both panels, samples are grouped on the x-axis as ALK-positive ALCL, ALK-negative ALCL, HIV-positive ALCL, EBER-negative DLBCL, EBER-positive DLBCL, and breast cancer. Microbial genera identified are listed on the y-axis.

**Abbreviations:** ALCL, anaplastic large cell lymphoma; ALK, anaplastic lymphoma kinase; DLBCL, diffuse large B-cell lymphoma; EBV, Epstein-Barr virus; HIV, human immunodeficiency virus.


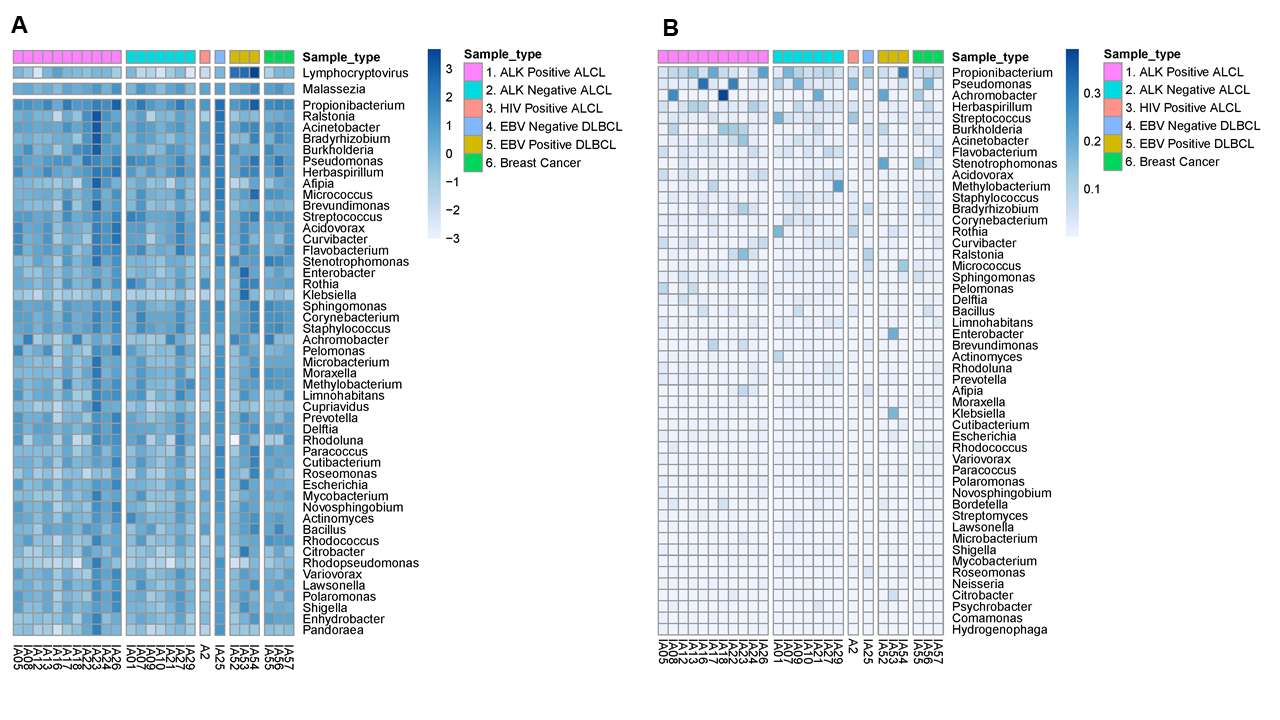


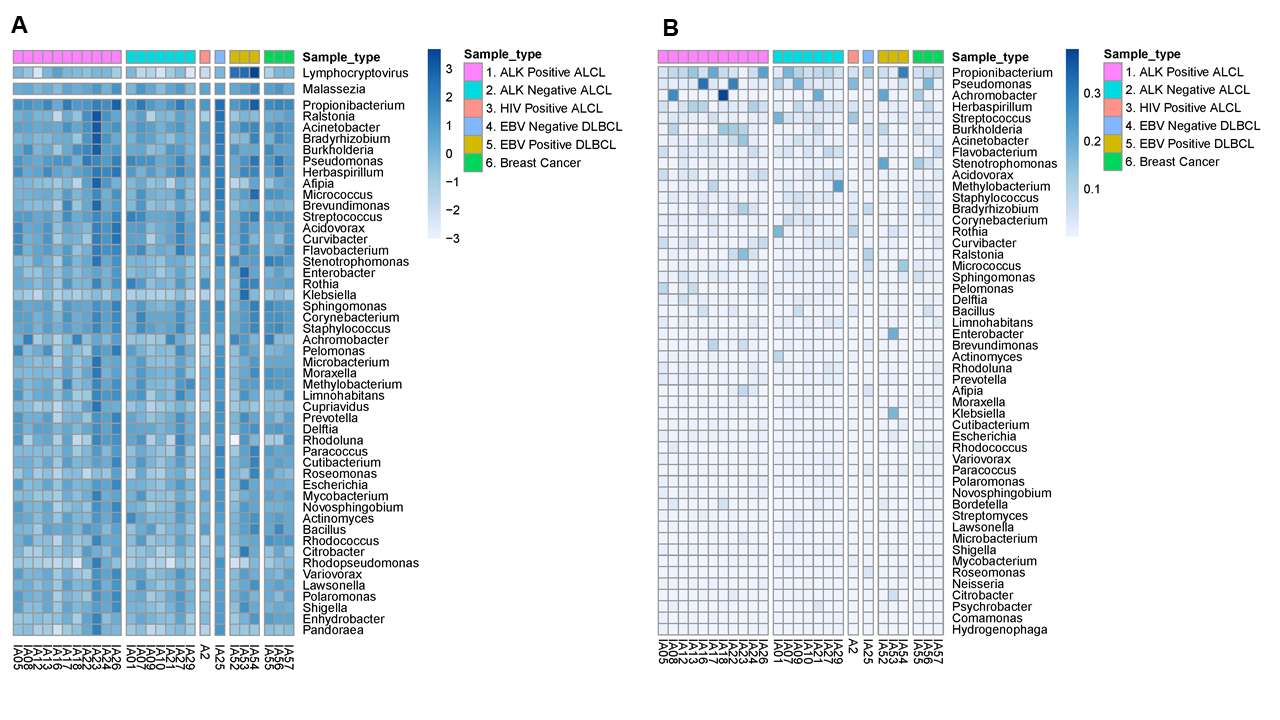


**Additional file 1: Figure S4.** Use of virID read-based approach to identify viral sequences associated with ALCL.

The figure highlights the findings of applying virID algorithm to classify unmapped reads following GATK-PathSeq using the read-based approach. Taxonomical classification of reads into viral genera after subjecting the unmapped reads directly to nucleotide (MegaBLAST) and translated amino acid (DIAMOND) searches against the reference databases are represented in panels (A) and (B), respectively. The units used are log_10_ reads per million human reads. Samples are grouped on the x-axis as ALK-positive ALCL, ALK-negative ALCL, HIV-positive ALCL, EBER-negative DLBCL, EBER-positive DLBCL, and breast cancer. Viral genera identified are listed on the y-axis.

**Abbreviations:** ALCL, anaplastic large cell lymphoma; ALK, anaplastic lymphoma kinase; DLBCL, diffuse large B-cell lymphoma; EBV, Epstein-Barr virus; HIV, human immunodeficiency virus.


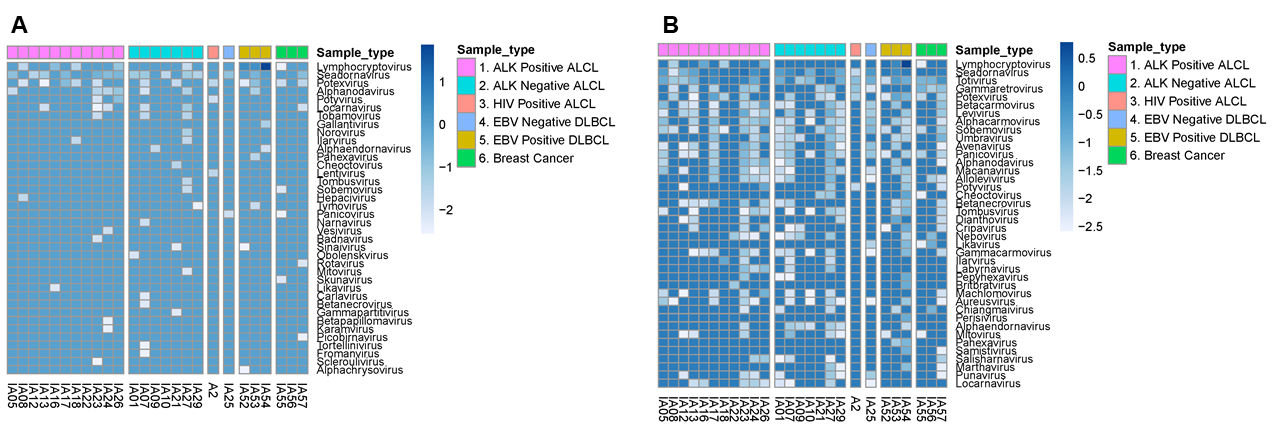


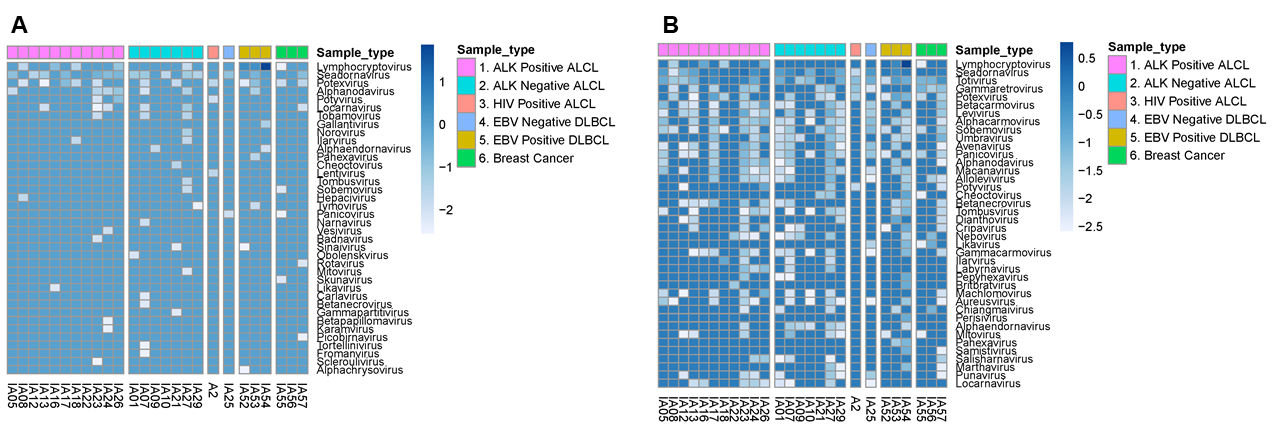


**Additional file 1: Figure S5.** The kmer enrichment approach to identify pathogen reads from unmapped GATK-PathSeq non-human reads.

The figure highlights the findings of kmer enrichment approach to classify unmapped reads following GATK-PathSeq. Taxonomical classification of reads into microbial genera after subjecting the kmer enriched reads to nucleotide (BLASTn) and translated amino acid (DIAMOND) searches against the reference databases are represented in panels (A) and (B), respectively. The units used are reads per million human reads. Samples are grouped on the x-axis as ALK-positive or ALK-negative ALCL. Microbial genera identified are listed on the y-axis.

**Abbreviations:** ALCL, anaplastic large cell lymphoma; ALK, anaplastic lymphoma kinase.


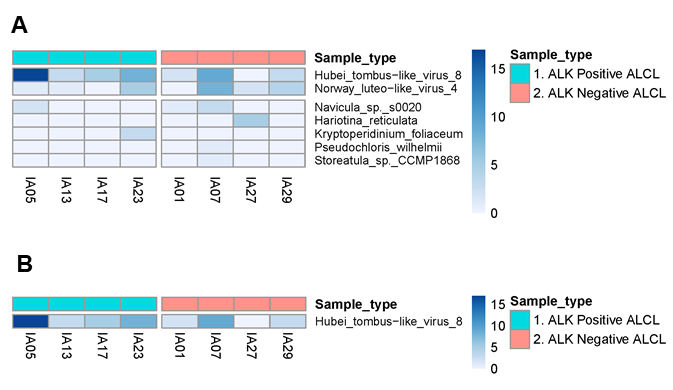


**Additional file 1: Table S1.** Quality control results for cases included in the analysis.

| **Study ID** | **Diagnosis** | **Calendar year of diagnosis** |  | **RIN** | **DV200 (%)** | **Input RNA, ng** | **Total RNA reads** |
| --- | --- | --- | --- | --- | --- | --- | --- |
| IA01 | ALCL | 2006 |  | 2.2 | 33 | 1000 | 700,086,166 |
| IA05 | ALCL | 2016 |  | 2.5 | 15 | 1000 | 669,116,640 |
| IA07 | ALCL | 2009 |  | 1.7 | 20 | 446 | 633,654,707 |
| IA08 | ALCL | 2001 |  | 2.6 | 6 | 1000 | 534,330,112 |
| IA09 | ALCL | 2006 |  | 2.5 | 8 | 500 | 171,373,470 |
| IA10 | ALCL | 2007 |  | 2.3 | 33 | 1000 | 755,274,654 |
| IA12 | ALCL | 2010 |  | 2.5 | 14 | 1000 | 745,090,362 |
| IA13 | ALCL | 2017 |  | 2.5 | 16 | 1000 | 672,095,290 |
| IA16 | ALCL | 2015 |  | 2.4 | 19 | 500 | 265,250,206 |
| IA17 | ALCL | 2010 |  | 1.5 | 18 | 500 | 429,775,068 |
| IA18 | ALCL | 2008 |  | 2.4 | 14 | 1000 | 517,609,404 |
| IA21 | ALCL | 2000 |  | 2.5 | 20 | 984 | 445,108,740 |
| IA22 | ALCL | 2004 |  | 2.4 | 17 | 500 | 126,614,668 |
| IA23 | ALCL | 2003 |  | 2.5 | 21 | 1000 | 643,062,668 |
| IA24 | ALCL | 2004 |  | 2.3 | 23 | 1000 | 479,742,598 |
| IA26 | ALCL | 2017 |  | 2.6 | 7 | 1000 | 600,126,966 |
| IA27 | ALCL | 2017 |  | 2.6 | 10 | 1000 | 662,259,012 |
| IA29 | ALCL | 2015 |  | 2.5 | 13 | 1000 | 628,460,348 |
| A2 | ALCL (HIV+) | 1997 |  | 1.0 | 28 | 500 | 136,912,782 |
| IA25 | DLBCL | 2006 |  | 2.5 | 13 | 1000 | 635,782,616 |
| IA52 | DLBCL | 2004 |  | 1.2 | 11 | 500 | 589,964,594 |
| IA53 | DLBCL | 2006 |  | 2.4 | 14 | 1000 | 133,611,902 |
| IA54 | DLBCL | 2002 |  | 2.5 | 13 | 500 | 144,899,144 |
| IA55 | Breast cancer | 1998 |  | 2.5 | 15 | 1000 | 486,047,792 |
| IA56 | Breast cancer | 1994 |  | 2.4 | 18 | 500 | 152,988,044 |
| IA57 | Breast cancer | 2006 |  | 2.5 | 25 | 1000 | 543,458,376 |

**Abbreviations:** ALCL, anaplastic large cell lymphoma; DLBCL, diffuse large B-cell lymphoma; DV, distribution value; RIN, RNA integrity number

**Additional file 1: Table S2.** Demographic and immunophenotypic features of cases and controls.

| Study ID | Diagnosis | Age at diagnosis (years) | Sex | Race | Diagnosis year | Primary site description | CD30 | CD2 | CD3 | CD20 | PAX5 | ALK1 | EBER |
| --- | --- | --- | --- | --- | --- | --- | --- | --- | --- | --- | --- | --- | --- |
| IA01 | ALCL | 75 | Female | White | 2006 | Right thigh mass | + | Golgi + | Weak + | − | − | − | − |
| IA05 | ALCL | 52 | Male | White | 2016 | Axillary lymph node | + | − | − | − | − | + | − |
| IA07 | ALCL | 78 | Male | White | 2009 | Axillary lymph node | + | Golgi + | − | − | − | − | − |
| IA08 | ALCL | 50 | Male | White | 2001 | Intra-abdominal lymph node | + | − | − | − | − | + | − |
| IA09 | ALCL | 40 | Female | White | 2006 | Inguinal lymph nodes | + | − | − | − | − | − | − |
| IA10 | ALCL | 35 | Female | White | 2007 | Cervical lymph nodes | + | + | + | − | − | − | − |
| IA12 | ALCL | 21 | Male | White | 2010 | T5-T8 vertebrae | + | Partial + | − | − | − | + | − |
| IA13 | ALCL | 36 | Female | White | 2017 | Soft tissue mass | + | Partial + | − | − | − | + | − |
| IA16 | ALCL | 45 | Male | Black | 2015 | Right thigh mass | + | + | + | − | − | + | − |
| IA17 | ALCL | 23 | Male | White | 2010 | Inguinal lymph nodes | + | − | − | − | − | + | − |
| IA18 | ALCL | 38 | Female | Unknown | 2008 | Axillary lymph node | + | − | − | − | − | + | − |
| IA21 | ALCL | 87 | Female | White | 2000 | Inguinal lymph nodes | + | Golgi + | Weak + | − | − | − | − |
| IA22 | ALCL | 36 | Male | White | 2004 | Inguinal lymph nodes | + | − | − | − | − | + | − |
| IA23 | ALCL | 43 | Female | White | 2003 | Intra-abdominal lymph node | Weak + | − | − | − | − | + | − |
| IA24 | ALCL | 60 | Male | White | 2004 | Small intestine | + | − | − | − | − | + | − |
| IA26 | ALCL | 55 | Male | White | 2017 | Cervical lymph nodes | + | − | − | − | − | + | − |
| IA27 | ALCL | 59 | Female | White | 2017 | Intra-abdominal lymph node | + | + | + | − | − | − | − |
| IA29 | ALCL | 37 | Female | White | 2015 | Posterior auricular lymph node | + | + | + | − | − | − | − |
| A2 | ALCL (HIV+) | 33 | Male | Unknown | 1997 | Nasal mass | + | + | + | − | Not tested | − | − |
| IA25 | DLBCL | 64 | Male | White | 2006 | Cervical lymph nodes | + | − | − | + | + | − | − |
| IA52 | DLBCL | 36 | Male | White | 2004 | Lung | Partial + | − | − | Variable + | Weak+ | − | + |
| IA53 | DLBCL | 65 | Male | White | 2006 | Small intestine | − | − | − | Variable + | Weak+ | − | + |
| IA54 | DLBCL | 66 | Male | White | 2002 | Intra-abdominal lymph node | − | − | − | Variable + | Weak+ | − | + |
| IA55 | Breast cancer | 42 | Female | White | 1998 | Breast |  |  |  |  |  |  |  |
| IA56 | Breast cancer | 50 | Female | White | 1994 | Breast |  |  | Not applicable | |  |  |  |
| IA57 | Breast cancer | 47 | Female | White | 2006 | Breast |  |  |  |  |  |  |  |

**Abbreviations:** ALCL, anaplastic large cell lymphoma; ALK1, anaplastic lymphoma kinase 1; CD, cluster of differentiation; DLBCL, diffuse large B cell lymphoma; EBER, Epstein-Barr virus-encoded small RNA; PAX5, paired box protein 5

**Additional file 1: Table S3**. Immunohistochemistry and in situ hybridization panel performed on ALCL and DLBCL tumor specimens.

| **Antibody** | **Clone** | **Company** | **Catalog #** |
| --- | --- | --- | --- |
| CD2 | MRQ-11 | Ventana | 760-4377 |
| CD3 | 2GV6 | Ventana | 790-4341 |
| CD20 | L26 | Ventana | 760-2531 |
| CD30 | Ber-H2 | Ventana | 790-4858 |
| PAX5 | SP34 | Ventana | 790-4420 |
| ALK1 | ALK01 | Ventana | 790-2918 |
| EBER-ISH | DNP-1 | Ventana | 760-1209 |

Abbreviations: ALCL, anaplastic large cell lymphoma; DLBCL, diffuse large B-cell lymphoma; EBER, Epstein-Barr virus-encoded small RNA; ISH, in situ hybridization.

**SUPPLEMENTARY METHODS**

***Selection of cases and controls:***

Hawaii and Iowa cancer registries have been a part of the National Cancer Institute’s Surveillance Epidemiology and End Results (SEER) program since 1973 and have residual and virtual tissue repositories. We identified 29 systemic anaplastic large cell lymphoma (ALCL) cases from the cancer registries using International Classification of Diseases for Oncology (3rd edition, ICD-O-3) morphology code 9714, and excluded cases with ICD-O-3 topography codes C44.x (skin) or C50.x (breast), or B-cell, pre-B-cell, or B-cell precursor cell types. Staff at the cancer registries then queried the tissue repository to identify cases for which formalin-fixed paraffin-embedded (FFPE) tissue blocks were available. Cases which were recently diagnosed and where the tissue was obtained from an excision biopsy were preferred in order to obtain adequate tumor tissue, better accuracy of the cancer registry diagnosis, and better RNA integrity.

For positive controls, we selected diffuse large B-cell lymphoma (DLBCL) cases arising in solid organ transplant recipients because most DLBCL cases in immunosuppressed individuals are associated with Epstein-Barr virus (EBV) infection. We leveraged a previous linkage between the US Scientific Registry of Transplant Recipients (SRTR) and cancer registries to identify DLBCL cases in transplant recipients.^1^ The Iowa state cancer registry is a participant in this Transplant Cancer Match Study. We identified 3 DLBCL cases linked to the transplant registry with adequate tissue available for the analysis. We selected 5 breast cancer cases as negative controls because breast cancer does not have a known viral etiology.

Because ALCL risk is highly elevated among immunosuppressed individuals, we sought to include ALCL cases among HIV-infected people or transplant recipients. We were able to obtain FFPE tissue from two ALCL cases that were diagnosed in HIV-infected persons at the Cook County Hospital (Chicago, Illinois) and Weill Cornell Medicine (New York, NY).

***Tissue processing and histopathology:***

To confirm the accuracy of diagnoses recorded in the cancer registries,^2^ we obtained two hematoxylin and eosin stained slides and thin (4 µm) tissue sections on charged slides from the cancer registry for each ALCL case and DLBCL control. Slides were put on an autostainer and went through the following steps: deparaffinization, rehydration, endogenous peroxidase activity inhibition, and antigen retrieval. Slides were incubated with the primary antibody followed by DISCOVERY anti-mouse HZ or anti-rabbit HQ and DISCOVERY anti-HQ-HRP detection system (Ventana, Tucson, AZ). Stains were visualized with DISCOVERY ChromoMap DAB kit and counterstained with hematoxylin (Ventana, Tucson, AZ). Two expert hematopathologists performed staining for CD30, B-cell markers (CD20, PAX-5), T-cell markers (CD2, CD3), anaplastic lymphoma kinase protein 1 (ALK1), and Epstein-Barr virus-encoded small RNAs (EBERs) to confirm the diagnosis of ALCL and DLBCL, characterized the immunophenotypic features, and confirmed the presence or absence of EBV in the tumors. The EBER in-situ hybridization (ISH) used the EBER 1 DNP Probe (#760-1209, Ventana) on the Ventana BenchMark Ultra (Ventana, Tucson, AZ) using the U ISH Open Probes Chromogenic V3 software with blue detection. Additional details on the immunohistochemistry and ISH panel are provided in **Additional file 1: Table S3**. Notably, one case that was reported as ALCL in the Iowa Cancer Registry (ID #IA25) was found to be misclassified DLBCL on immunohistochemistry but retained in the study as an EBER-negative DLBCL control arising in the general population, besides the three EBER-positive DLBCL controls in transplant recipients.

In addition, up to ten 20 µm sections in thickness were obtained from each tumor (ALCL, DLBCL, and breast cancer) and placed into a sterile RNase free microfuge tube for RNA extraction and sequencing. The histotechnicians preparing these sections took precautions to avoid contamination of the sections by using a new blade for each tumor sample and wiping down the apparatus with RNase decontamination wipes.

***RNA extraction, library preparation, and sequencing:***

FFPE sections were simultaneously deparaffinized and digested using 400µL molecular grade mineral oil (Millipore-Sigma) and 255 Buffer ATL (Qiagen) with 45µL of proteinase K (Qiagen). Samples were incubated overnight at 65°C in a shaking heat block. Samples were spun at max speed in a tabletop centrifuge for one minute to separate the organic and aqueous phases. Depending on visible remaining tissue, some samples were subjected to one or two additional two-hour long digests by the addition of 25µL new proteinase K. 150µL of the remaining lysate was moved to a new tube. 250µL of buffer PKD was added and vortexed to mix. The remainder of RNA extraction was carried out using RNeasy FFPE Kit (Qiagen) and the manufacturer’s protocol. RNA quantity and quality were assessed by spectrophotometry and the 2100 Bioanalyzer system (Agilent). Total RNA libraries were prepared using the KAPA RNA HyperPrep Kit (Roche). Five samples (IA01, IA53, IA57, IA07, and IA17) were sequenced on a NextSeq 500 (Illumina) and the remainder on a NovSeq 6000 (Illumina). Forward and reverse read lengths were 150 bp. To detect potentially low viral loads, the targeted number of reads was 600 million per sample, and most samples had 400-800 million reads due to variation in loading estimates (**Additional file 1: Table S1**). Samples with fewer than 400 million reads had lower RNA input volumes due to lower sample quality. Libraries could be successfully prepared for 19 ALCL cases (including one case in an HIV-infected person), 4 DLBCL controls (including IA25), and 3 breast cancer controls. These cases/controls were included in the final metagenomic analysis.

***Metagenomic analysis:***

GATK-PathSeq:

GATK-PathSeq is a customizable computational tool that uses a comprehensive and systematic subtraction approach to remove human host reads in sequencing data followed by alignment to a database of microbial reference genomes.^3^ Detected organisms are scored based on taxonomic abundance, and unmapped sequences can then be further examined for new and novel organisms. Details on the functioning of this pipeline have been previously described.^3^ For this study, the standard GATK-PathSeq microbial reference database, based on RefSeq, was used. GATK-PathSeq is freely available at <https://gatk.broadinstitute.org/hc/en-us>.

virID:

virID can operate in two modes, assembly and read-based. In assembly mode, input reads are assembled into contigs using the rnaSPAdes assembler.^4^ These contigs are then queried against the NCBI “nt” nucleotide reference database using MegaBLAST^5^ and against the RefSeq nonredundant protein reference database using DIAMOND.^6^ Because one contig may be assigned to multiple taxa, the last-common-ancestor of the top assignments for each contig is taken. To quantify the resultant taxa, reads are mapped to the contigs using BWA-MEM.^7^ In read-based mode, reads are directly queried with MegaBLAST and DIAMOND. This is particularly helpful when the assembly efficiency of some samples is poor. To visualize virID outputs, each read is assigned to every member of the taxonomic lineage of the read (read-based mode) or contig (assembly mode), resulting in cumulative counts at each taxonomic level. More information on the virID pipeline has been published elsewhere.^8^ virID is freely available at: <https://github.com/jnoms/virID/>.

Kmer-enrichment approach with virKMER:

In this approach, continuous 20 base pair sequences (20mers) were extracted from input ALCL samples, control tumor samples, and the viral nucleotide database RefSeq.^9^ These 20mers that were shared by at least two ALCL samples and the RefSeq viral database, but not the control tumor samples, were identified. The reads that contain these kmers were then classified using BLASTN. virKMER is publicly available at <https://github.com/jnoms/virKMER>.

**Additional references for the Supplementary Methods:**

1. Engels EA, Pfeiffer RM, Fraumeni JF, Jr., et al. Spectrum of cancer risk among US solid organ transplant recipients. *Jama*. 2011;306(17):1891-1901.

2. Mahale P, Weisenburger DD, Kahn AR, et al. Anaplastic large cell lymphoma in human immunodeficiency virus-infected people and solid organ transplant recipients. *Br J Haematol*. 2020;(In press).

3. Walker MA, Pedamallu CS, Ojesina AI, et al. GATK PathSeq: a customizable computational tool for the discovery and identification of microbial sequences in libraries from eukaryotic hosts. *Bioinformatics*. 2018;34(24):4287-4289.

4. Bushmanova E, Antipov D, Lapidus A, Prjibelski AD. rnaSPAdes: a de novo transcriptome assembler and its application to RNA-Seq data. *Gigascience*. 2019;8(9).

5. Camacho C, Coulouris G, Avagyan V, et al. BLAST+: architecture and applications. *BMC Bioinformatics*. 2009;10:421.

6. Buchfink B, Xie C, Huson DH. Fast and sensitive protein alignment using DIAMOND. *Nat Methods*. 2015;12(1):59-60.

7. Li H. Aligning sequence reads, clone sequences and assembly contigs with BWA-MEM. <https://arxiv.org/abs/1303.3997>. Accessed April 14, 2020.

8. Nomburg J, Bullman S, Chung SS, et al. Comprehensive metagenomic analysis of blastic plasmacytoid dendritic cell neoplasm. *Blood Adv*. 2020;4(6):1006-1011.

9. Pruitt KD, Tatusova T, Maglott DR. NCBI reference sequences (RefSeq): a curated non-redundant sequence database of genomes, transcripts and proteins. *Nucleic Acids Res*. 2007;35(Database issue):D61-65.
